# COVID-19 victimization experience and university students’ smartphone addiction: the mediating role of emotional intelligence

**DOI:** 10.1186/s12889-023-16355-7

**Published:** 2023-07-22

**Authors:** Hongxia Chen, Hong-xin Zhang

**Affiliations:** 1 School of Marxism, Zhoukou Normal University, 466001 Wenchang Road, Zhoukou City, Henan Province China; 2 Institute for Public Health, Zhoukou Normal University, 466001 Wenchang Road, Zhoukou City, Henan Province China

**Keywords:** Post-COVID-19 era, COVID-19 victimization experience, Smartphone addiction, Emotional intelligence, University students

## Abstract

**Objectives:**

During the post-COVID-19 era, everyone has the risk of contracting the virus and becoming the victims of COVID-19. Examining the relationship between the COVID-19 victimization experience and its effects is more urgent. The aim of present study is to propose a mediation model to investigate the association between COVID-19 victimization experience and smartphone addiction, and the mediating role of emotional intelligence.

**Methods:**

A online questionnaire including the COVID-19 Victimization Experience Scale, the Smartphone Addiction Scale, and the Emotional Intelligence Scale among Chinese university students, were employed in this study. Finally, 1154 valid questionnaires were collected. The reliability and confirmatory factor analysis results showed that all three scales had good reliability and validity.

**Results:**

Structural Equation Model (SEM) demonstrated that COVID-19 victimization experience significantly predicted smartphone addiction in university students, and emotional intelligence partially mediated the association between COVID-19 victimization experience and smartphone addiction. Bootstrap results furthermore tested the rigor of the mediating effect.

**Conclusion:**

COVID-19 victimization experience was a important variables in predicting university students’s martphone addiction, and emotional intelligence was a protective factor in decreasing the negative effect of COVID-19 victimization experience on addictive behaviors. It is suggested that instructors should integrate emotional intelligence training programs into mental health courses so as to improve students’ emotional intelligence ability.

## Introduction

Smartphone addiction, also known as “problematic smartphone use”, refers to the loss of self-control resulting from the overuse of smartphone phones and causes difficulties in everyday life [1]. Previous studies have confirmed that smartphone addiction can cause serious harm to individuals in several aspects, such as inability to express emotions, depression, anxiety, sleep disorders, social dysfunction, and even suicide [2–4]. Therefore, smartphone addiction has attracted considerable attention theoretically and practically. Additionally, due to the particular psychological characteristics and social interactions, university students are more prone to indulge in smartphone use, resulting in smartphone addiction.

With the outbreak of COVID-19, the frequency and dependence of university students on smartphones has being increased during the COVID-19 pandemic, which might cause the risk of smartphone addiction and negatively affect their academic performance and mental health [5–7]. Past findings has suggested that the fear of COVID-19, the COVID-19 perceived risk, and anxiety regarding COVID-19 infection are all considered as significant positive predictors of smartphone addiction [8–11]. Another study also have confirmed that trauma exerted a significant predictive effect on smartphone addiction among university students [12]. Since December 6, 2022, Chinese government had changed its previous zero COVID policy, particularly no lockdown measures and no compulsory NAT (nucleic acid test), which means that everyone in China may contract the virus and become the victims of COVID-19. In this sense, examining the relationship between the COVID-19 victimization experience and smartphone addiction and its potential mechanism is more urgent and important. Also, less existing research has reported that emotional intelligence is likely a significant predictor of smartphone addiction [13–15]. However, the potential mechanistic implications in context of COVID-19 remain largely unknown. To the best of our knowledge, no studies have investigated the mediating emotional intelligence in the association between COVID-19 victimization experience and smartphone addiction.

Therefore, the present study attempted to fill the gap by examining the mediating role of emotional intelligence in the association between COVID-19 victimization experience and smartphone addiction. According to the social cognitive theory (SCT) [16], the present study targeted at university students under lockdown in China, regarding the COVID-19 victimization experience as an environmental factor, emotional intelligence as an individual factor, and smartphone addiction as a behavioral factor, comprehensively exploring how the COVID-19 victimization experience affects their smartphone addiction behavior. The present study aimed to examine the psychological mechanism between the COVID-19 victimization experience and smartphone addiction. The findings of this empirical study can help understand the crucial factors affecting university students’ smartphone addiction, thereby providing new sights for educators on how to reduce the risk of smartphone addiction among university students when confronting the ongoing and possible future pandemics.

### Social cognitive theory

According to Bandura [16] who proposed the theory, social cognitive theory (SCT) affords a model in which three factors, i.e., external environmental, intra-individual and behavioral factors is not isolate but could interact with each other. SCT have applied in the filed of addictive behavior. For example, based on SCT, in a sample of 277 young smartphone users in China, Wu et al. (2013) [17] indicated that those who spent more time on social networks (environmental factor) also reported higher addictive tendencies (behavioral factor), three psychological variables (intra-individual factors), i.e., outcome expectancies and impulsivity, Internet self-efficacy were correlated with addictive tendencies, accounting for 23% of the variance in addictive tendencies. Similarly, on the basis of SCT, Yang [18] constructed a mediation model of self-efficacy and self-control (personal cognitive factors) affecting Internet addiction addiction in adolescents (behavior factors) through social support (environmental factors). More recently, in a study conducted by Pan et al. [19], during the COVID-19 lockdown in China, investigated the influence of the COVID-19 perceived risk (environmental factor) on Internet addiction (behavioral factor) among 690 college students through difficulties in emotion regulation (personal cognitive factors).

With regard to smartphone addiction, SCT was also applied in current studies. For instance, Lian et al. [20] linked two negative environmental factors, i.e., parental rejection and overprotection parenting style, with a higher degree of Smartphone addiction (personal factors); they found that personal virtue factors, i.e., relationship, vitality, and conscientiousness, mediated the association between parenting style and Smartphone addiction among college students. Similarly, in a sample of 2,172 high school students, based on SCT, Cheng et al. [21] revealed that the parent–child relationship (environmental factor) was negatively related both to smartphone addiction (behavioral factor) and loneliness (personal factors). Specifically, when the parent–child relationship improves, loneliness will ease and smartphone addiction will accordingly lessen. More recently, on the basis of SCT, in a sample of 840 Chinese college students, Chen et al. [22] regarded COVID-19 victimization experience as an environmental factor and regarded mobile phone addiction as a behavioral factor; their findings revealed that future anxiety as a personal factor fully mediated the association between COVID-19 victimization experience and mobile phone addiction and mindfulness as a personal factor moderated the effect of COVID-19 victimization experience on the college students’ future anxiety.

Therefore, based on SCT and above related studies, the present study will consider COVID-19 victimization experience as an environmental factor, emotional intelligence as an individual factor and smartphone addiction as a behavioral factor. In addition, the mediating role of emotional intelligence in the COVID-19 victimization experience—smartphone addiction link were examined.

### COVID-19 victimization experience and smartphone addiction

COVID-19 victimization experience refers to one’s negative thoughts associated with the coronavirus event and psychological trauma associated with the disaster breakout process [23]; it consists of two parts: catastrophic cognition and trauma symptoms. The former was described as when confronting the disaster events individuals merely focus on the negative aspects; the latter were described as those resulting from individuals experiencing, witnessing, or facing various stressful events related to the occurrence of the coronavirus, and negative psychological symptoms, such as tension, fear of infection, insomnia, and moodiness may develop.

Previous Studies have consistently reported that adults with disaster victimization experiences may develop mental health, such as depression, emotional distress, and worsening psychological symptoms [24–28]. More specifically, students with trauma exposure and negatively cognitive cognition during and after the disaster events reported the worst mental health. For example, a sample of 19,861 university students from America, Artime et al. [29] founded that both interpersonal violence and service-members with combat-related trauma could impact university students’ mental health and academic functioning. More recently, in a sample of 384 university students in Wuhan from China, where the COVID-19 first broke out, Yang et al. [23] confirmed that the COVID-19 victimization experience was a negative predictor of university students’ mental health; positive thinking and resilience could mediate the relationship between COVID-19 victimization experience and mental health. Meanwhile, a recent study indicated that university students are one of the most affected groups by the COVID-19 pandemic [30]. Thus, examining the COVID-19 victimization experience and its effects among university students has gained considerable attention from scholars.

As for the relationship between the university students’ COVID-19 victimization experience and their smartphone addiction, there are fewer directly studies. However, some recent studies, in the context of the COVID-19 and other trauma events, have identified some potential factors causing smartphone or Internet addiction [5–8]. One of these studies, conducted in Turkey among 773 adults, Kayis et al. [8] found that the fear of COVID-19 influenced mental wellbeing via loneliness and smartphone addiction. Another empirical study including 550 users of social media in Germany demonstrated that the burden caused by COVID-19, such as experiencing control loss over their daily activities and anxiety symptoms, could exert a significant positive prediction effect on addictive social media use behavior [31]. Similarly, in a study conducted by Liang et al. [12] among 263 Chinese college students noted that childhood trauma was associated with high smartphone addiction; adult attachment partially mediated the relationship between childhood trauma and smartphone addiction. In Zwilling’ [32] two wave of study conduced during and after the COVID-19 Pandemic among 207 young adults in Israeli, loneliness, the need for social interaction, sleep hours, fear of losing phone access, and stress all affected problematic smartphone use. More recently, a study conduced by Chen et al. [22], in a sample of 840 Chinese college students, showed that COVID-19 victimization experience significantly predicted college students’ mobile phone addiction and future anxiety fully mediated the association.

Additionally, the sense of isolation due to the lockdown policies during the Covid-19 period limited the opportunities of individuals to receive information. With the increasing epidemic and depending upon the severity of their problems, individuals experiencing suspicious COVID-19 pandemic-related symptoms would often seek information via their smartphone devices to protect themselves against the disease and to check out whether the symptoms they perceived are symptoms of the COVID-19. This situation, termed as ‘cyberchondria’ was a situation that triggered excessive health-related Internet use [33, 34]. In the context of Covid-19, the occurrence of cyberchondria and the association between cyberchondria and smartphone addiction was also reported by some studies [35, 36]. For instance, according to Yam et al. [36], cyberchondria severity could have both moderating and mediating effect in the link between smartphone addiction and the fear of COVID-19.

Thus, based on the aforementioned findings, the present study proposed the first primary hypothesis as follows:

- Hypothesis 1. University students’ COVID-19 victimization experience would exert a significant positive prediction effect on smartphone addiction.

### The mediating role of emotional intelligence

Emotional intelligence refers to the ability to perceive, appraise, use, and regulate one’s own and others’ emotions, and subsequently use this information to guide one’s thinking and actions [37, 38]; it is a key determinant of physical and mental health, psychological adjustment, quality of life and success in a variety of occupational settings [39]. As to students, emotional intelligence could negatively correlated with burnout and anxiety levels, and positively predict psychological health and academic performance [40]. In addictive behavior, previous studies also have showed that individuals with poor emotional intelligence are more likely to associate with problem behaviors. A systematic review on emotional intelligence and behavioral addictions revealed that a lower level of EI is associated with more intensive smoking, alcohol use, and illicit drug use [14].

The importance of emotional intelligence as a protective factor for emotional experiences, negative emotions, mental health, and work performance in the context of work place was examined by prior studies during the COVID-19 pandemic [41–43]. With respect to Internet and/or smartphone addiction, there are few studies that deal explicitly with addictive behavior and emotional intelligence [13, 15]. For example, Engelberg and Sjöberg’s [39] found that individuals with emotional intelligence tended to be lonely, had deviant values and frequently used the Internet. According to Beranuy et al. [15], apart from psychological distress, perceived emotional intelligence could also significantly predict maladaptive use of Internet and mobile phone. Likewise, Van Deursen et al. [13] emphasized that self-regulated individuals who are able to understand emotions and regulate feelings are better adjusted psychologically, are more unlikely to adversely affected by smartphone addiction. Mascia et al. [44] suggested that adolescents’ emotional intelligence negatively influenced addictive smartphone behavior, which in turn influenced their well-being and quality of life. More recently, in a one-year prospective study among 500 college student, Tsai et al. [45] indicated that difficulties in emotion regulation, a component of emotional intelligence, could predict the incidence of Internet addiction. Overall, these facts suggest that university students with lower levels of emotional intelligence may possess a higher risk of developing smartphone addiction.

Additionally, some empirical studies also found that individuals’ levels of emotional intelligence can be altered by high levels of COVID-19 victimization experience. For instance, using a sample of Children Aged 9–10, Martín-Requejo and Santiago-Ramajo found a reduced emotional intelligence due to the COVID-19 Pandemic Lockdown [46]. More recently, in a sample of 972 Czech citizens, according to Hajkova et al. [47], individuals experiencing COVID-19 related symptoms such as depression and anxiety tend to report experiencing a range of emotional, cognitive, and behavioral changes. Moreover, previous findings have suggested that emotional intelligence often plays an essential mediating role. For example, a study conduced by Kürsad et al. [48], in a sample of 297 university students in Turkey, found that emotion regulation difficulties, a component of emotional intelligence, had a full mediating role in the relationship between social anxiety and problematic internet use. Similarly, in a sample of 690 Chinese college students during the COVID-19 lockdown, Pan et al. [19] reported that the COVID-19 perceived risk was significantly positively associated with Internet addiction, and difficulties in emotion regulation partially mediated the relationship between COVID-19 perceived risk and Internet addiction (indirect effect value was 0.051). More recently, in a sample of 241 medical students in Malaysia, Yusoff et al. [40] found that psychological distress significantly increased burnout level, while emotional intelligence had a significant mediating effect on reducing burnout. Thus, the present study proposed the second hypothesis as follows:

- Hypothesis 2. Emotional intelligence would have a mediating effect on the relationship between COVID-19 victimization experience and smartphone addiction among university students.

### The present study

**Fig. 1:**
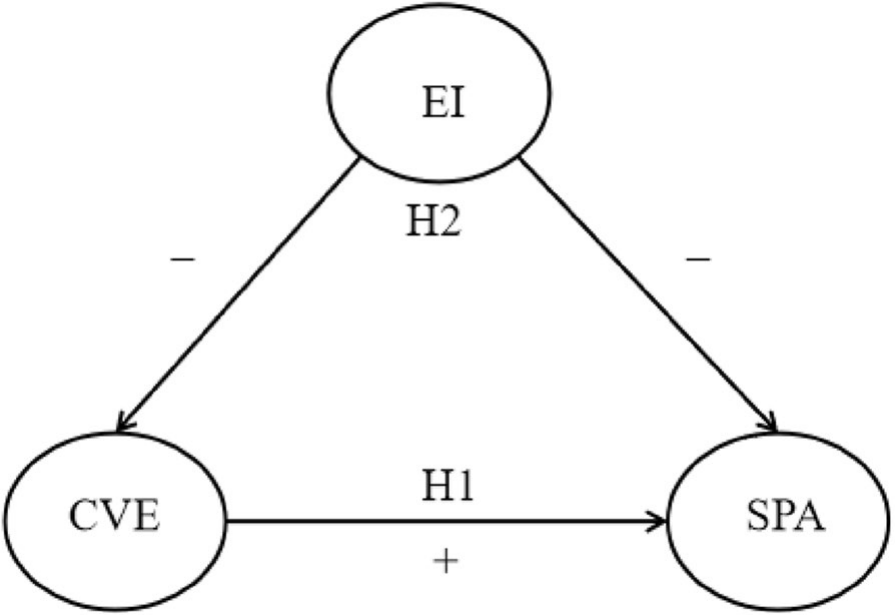
The proposed mediation model. Note: CVE, COVID-19 victimization experience; EI, emotional intelligence; SPA, smartphone addiction

On the basis of the above theoretical analysis and literature discussion, this study constructed a mediated model to examine the relationship between COVID-19 victimization experience and smartphone phone addiction, which is shown in Fig. 1:

## Materials and methods

### Participants

The Ethics Committee of the Zhoukou Normal University (ZKNMREB-2022–0912) approved the present study. The procedure of study was conducted in accordance with the Declaration of Helsinki [49], and all participants voluntarily filled out questionnaires. The data in the present study were collected and analyzed anonymously.

Participants were enrolled by means of convenience sampling. A questionnaire survey was conducted at two universities from China, one in Zhoukou city, and the other in Shenyang city. Questionnaires were distributed on the online, since no questionnaire was submitted until all items were completed, so there was no uncompleted questionnaire, and 1,223 questionnaires were gathered. The entire questionnaire took about 15 min. After excluding 69 invalid questionnaires, because the answering time was too low (i.e., completed in lower than 120 s), finally 1,154 valid questionnaires were collected, with an effective rate of 94.4%.

### Measures

#### The COVID-19 victimization experience scale

To measure university students’ COVID-19 victimization experiences, the present study used the COVID-19 Victimization Experience Scale (CVES-8) developed by Yang et al. [23]. The eight-item scale consists of two dimensions: catastrophic cognition (e.g., I think that what happened to me was the worst) and trauma symptoms (e.g., My body often feels tense). The responses are rated on a 5-point Likert scale, ranging from 1 (strongly disagree) to 5 (strongly agree), with higher scores revealing higher levels of traumatic experiences of the COVID-19 pandemic.

#### The smartphone addiction scale

To measure the level of smartphone addiction among university students, the Smartphone Addiction Scale (SAS-10) developed by Kwon et al. [50] was used in this study. The responses are rated on a 5-point Likert scale ranging from 1 (not at all) to 5 (always), with higher scores indicating higher levels of smartphone addiction. The sample item is: “I Won’t be able to stand not having a smartphone”.

#### The emotional intelligence scale

Emotional intelligence was assessed with the Wong and Law Emotional Intelligence Scale (WLEIS-16) [34]. This scale is a 16 items self-report measure with a 5-point Likert scale ranging from 1 (strongly disagree) to 5 (completely agree), with higher scores suggesting higher levels of emotional intelligence, including four dimensions: (1) Self-emotion appraisal (SEA), with 4 items (e.g., I really understand what I feel); (2) Others’ emotion appraisal (OEA), with 4 items (e.g., I am a good observer of others’ emotions); (3) Use of emotion (UOE), with 4 items (e.g., I am a self-motivated person); and (4) Regulation of emotion (ROE), with 4 items (e.g., I have good control of my own emotions). The Chinese version has shown satisfactory psychometric properties [51].

### Procedure and data analysis

The statistical procedure for this study ran as follow. First, Harman’s single factor was used to test the common method variance (CMV). Then, participants’ profile, descriptive statistics and correlation analysis of the variables was used with SPSS 21.0. Second, according to Anderson and Gerbing (1988) [52] suggestion, Confirmatory Factor Analysis (CFA) was performed to test the structural validity of the measurement model. Then the fit and path coefficients of the hypothesized mediation model were assessed by the Structural Equation Model (SEM) with AMOS 21.0, not least because it could test relationships between many factors in a hypothesized model simultaneously [53]. Finally, for the rigor of the mediating effect, the bias-corrected nonparametric percentile Bootstrap method with 5,000 times resampling was used. According to Preacher and Hayes [54], compared with the traditional causal steps, the method of Bootstrapping has shown greater statistical power.

### Common Method Variance (CMV) test

Before formal analysis, since all variables were measured with self-reported scale among the same participants, there may be the Common Method Variance (CMV) problem. To deal with this problem, Harman’ s one-factor test was used. More specifically, the Bartlett test of sphericity reached significance (*p* < 0.001), and Unrotated factor analysis indicated that the Kaiser–Meyer–Olkin (KMO) was 0.911 (more than 0.8). Furthermore, a total of 7 factors were extracted from the factor analysis, and the explanatory power of the first factor was 25.223%, which didn’t overpass 50% threshold [53], indicating that the CMV problem did not affect the study results.

## Results

### Participants’ profile

A total of 1154 participants were enrolled in this study, the results of participants’ profile were shown in Table 1. As shown, 525 (45.5%) were male students and 629 (54.5%) were female students. As to age, 40 (3.5%) were 17-year-old, 369 (32%) were 18, 328 (28.4%) were 19, and 417 (36.1%) were 20. As regard to grade, the sample included 561 (48.6%) freshmen, 281 (24.4%) sophomores, 282 (24.4%) juniors and 30 (2.6%) seniors. In terms of major, 425 (36.8%) were arts and humanities, 320 (27.7%) were science and 409 (35.4%) were engineering.

**Table 1:** Demographic profile of participants

Item	Category	Frequency	Percent
Gender	Male	525	45.5%
	Female	629	54.5%
Age	17	40	3.5%
	18	369	32%
	19	328	28.4%
	20	417	36.1%
Grade	Freshman	561	48.6%
	Sophomore	281	24.4%
	Junior	282	24.4%
	Senior	30	2.6%
Major	Arts and Humanities	425	36.8%
	Science	320	27.7%
	Engineering	409	35.4%

### Reliability and validity assessment of measurement model

#### The COVID-19 victimization experience scale

The results of confirmatory factor analysis of the COVID-19 Victimization Experience Scale (CVES-8) was displayed in Table 2. According to the results, the factor loadings of CVES-8 were ranging from 0.671 to 0.865 (more than 0.5), the composite reliability (CR) values were ranging from 0.810 to 0.884 (more than 0.6), and the average variance extracted (AVE) values were between 0.518 and 0.658 (more than 0.5). All the values went beyond the standard value, suggesting the high convergent validity of CVES [55, 56]. Therefore, the convergent validity of CVES was appropriate. The Cronbach’sαof each dimension was between 0.810 and 0.883, greater than 0.6, indicating the excellent reliability [57].

**Table 2:** CFA results of CVES-8

Dimension	NO	Factor Loading	CR	AVE	Cronbach’s α
Catastrophic cognition	1	0.711	0.884	0.658	0.883
	2	0.813			
	3	0.846			
	4	0.865			
Trauma symptoms	1	0.775	0.810	0.518	0.810
	2	0.671			
	3	0.675			
	4	0.751			

*CR* composite reliability, *AVE* average variance extracted

#### The smartphone addiction scale

The results of confirmatory factor analysis of the Smartphone Addiction Scale (SAS-10) was displayed in Table 3. According to the results, the factor loadings of SAS-10 were ranging from 0.471 to 0.715 (more than 0.5), the CR value was 0.849 (more than 0.7), and the AVE value was 0.364 (less than 0.5). Even though the AVE is less than the threshold of 0.5, however, according to Fornell and Laecker [58], if the CR value meeting the criteria (i.e., greater than 0.6), the convergent validity of the scale is still acceptable. The Cronbach’sαwas 0.871, greater than 0.7, indicating the excellent reliability [57].

**Table 3:** CFA results of SAS-10

Dimension	NO	Factor Loading	CR	AVE	Cronbach’s α
Smartphone addiction	1	0.529	0.849	0.364	0.847
	2	0.606			
	3	0.536			
	4	0.574			
	5	0.711			
	6	0.715			
	7	0.603			
	8	0.471			
	9	0.653			
	10	0.586			

*CR* composite reliability, *AVE* average variance extracted

#### The emotional intelligence scale

The results of confirmatory factor analysis of the Wong and Law Emotional Intelligence Scale (WLEIS-16) was displayed in Table 4. According to the results, the factor loadings of WLEIS-16 were ranging from 0.631 and 0.886 (more than 0.5), the CR values were between 0.849 and 0.892 (more than 0.7), and AVE values were ranging from 0.587 to 0.676 (more than 0.5). All the values exceeded the standard value, indicating the high convergent validity of the EIS-16 [55, 56]. The Cronbach’s α of each dimension was between 0.844 and 0.889, greater than 0.7, indicating the excellent reliability [57].

**Table 4:** CFA results of WLEIS-16

Dimension	NO	Factor Loading	CR	AVE	Cronbach’s α
Self-emotion appraisal	1	0.710	0.859	0.606	0.856
	2	0.817			
	3	0.851			
	4	0.726			
Others’ emotion appraisal	1	0.768	0.892	0.676	0.889
	2	0.886			
	3	0.881			
	4	0.744			
Use of emotion	1	0.631	0.849	0.587	0.844
	2	0.817			
	3	0.807			
	4	0.794			
Regulation of emotion	1	0.786	0.878	0.643	0.877
	2	0.805			
	3	0.767			
	4	0.848			

*CR* composite reliability, *AVE* average variance extracted

### Discriminant validity

To test the discrimination, according to Fornell and Larckers’ suggestion, the square root of AVE was used [58]. As displayed in Table 5, the square root of AVE of each dimension was greater than the correlation coefficient of each dimension, indicating the high discriminant validity.

**Table 5:** Discriminant validity

Dimension	M	SD	1	2	3	4	5	6	7
1.CVE-CC	2.433	0.894	**0.811**						
2.CVE-TS	2.448	0.859	0.721***	**0.720**					
3.EI-SEA	3.676	0.660	-0.265***	-0.288***	**0.778**				
4.EI-OEA	3.636	0.692	-0.085**	-0.149***	0.521***	**0.822**			
5.EI-UOE	3.528	0.705	-0.211***	-0.218***	0.555***	0.477***	**0.766**		
6.EI-ROE	3.504	0.723	-0.130***	-0.211***	0.486***	0.382***	0.511***	**0.802**	
7.SPA	2.887	0.694	0.235***	0.287***	-0.162***	-0.138***	-0.199***	-0.224***	**0.603**

*n* = 1154; ** *p* < 0.01, *** *p* < 0.001; *M* mean, *SD* standard deviation, *CVE-CC* catastrophic cognition, *CVE-TS* trauma symptoms, *EI-SEA* self-emotion appraisal, *EI-OEA* Others’ emotion appraisal, *EI-UOE* use of emotion, *EI-ROE* regulation of emotion, *SPA* smartphone addiction. The bold numbers in the diagonal are the square root of AVE (AVE = average variance extracted); numbers in the lower diagonal denote the correlation coefficients

### Descriptive statistics and correlation analysis of main variables

Descriptive statistics and correlation analysis were shown in Table 6. The statistical results showed that university students had a mean level of COVID-19 victimization experience (mean = 2.441), smartphone addiction (mean = 2.887), and emotional intelligence (mean = 3.586) in the present study. In addition, the results indicated that the COVID-19 victimization experience and smartphone addiction had significantly positive correlation (*r* = 0.281, *p* < 0.001); emotional intelligence and the COVID-19 victimization experience had significantly negative correlation (*r* = -0.265, *p* < 0.001); emotional intelligence and smartphone addiction had significantly negative correlation (*r* = -0.231, *p* < 0.001).

**Table 6:** Descriptive statistics and correlation analysis

Variable	M	SD	CVE	EI	MPA
CVE	2.441	0.813	1		
EI	3.586	0.545	-0.265***	1	
SPA	2.887	0.694	0.281***	-0.231***	1

*n* = 1154; *** *p* < 0.001; *M* mean, *SD* standard deviation, *CVE* COVID-19 victimization experience, *EI* emotional intelligence, *SPA* smartphone addiction

### Structural model

To test the proposed mediation model in Fig. 1, the present student conduced a second-order SEM analysis. The goodness of fit indices for the COVID-19 victimization experience-smartphone addiction relationship and the mediating effects of emotional intelligence are summarized in Table 7. Specifically, the selected absolute, incremental and parsimonious model-fit indices (RMSEA, GFI, CFI, TLI, TLI, NFI and CMIN/DF) all satisfied the criteria, indicating a good fit of the proposed model.

**Table 7:** Summary of criteria, estimated values, and model fit measures

Model Fit Measures	Name of index	Criteria	Estimated Values	Fit
Absolute fit	RMSEA	< 0.8	0.052	Yes
	GFI	> 0.9 or > 0.8	0.894	Yes
Incremental fit	CFI	> 0.9 or > 0.8	0.920	Yes
	TLI	> 0.9 or > 0.8	0.913	Yes
	NFI	> 0.9 or > 0.8	0.897	Yes
Parsimonious fit	CMIN/DF	< 3 or < 5	4.088	Yes

*RMSEA* root mean square of error approximation, *GFI* goodness of fit index, *CFI* comparative fit index, *TLI* Tucker-Lewis index, *NFI* normed fit index, *CMIN/DF* Chi-square/degree of freedom

**Fig. 2:**
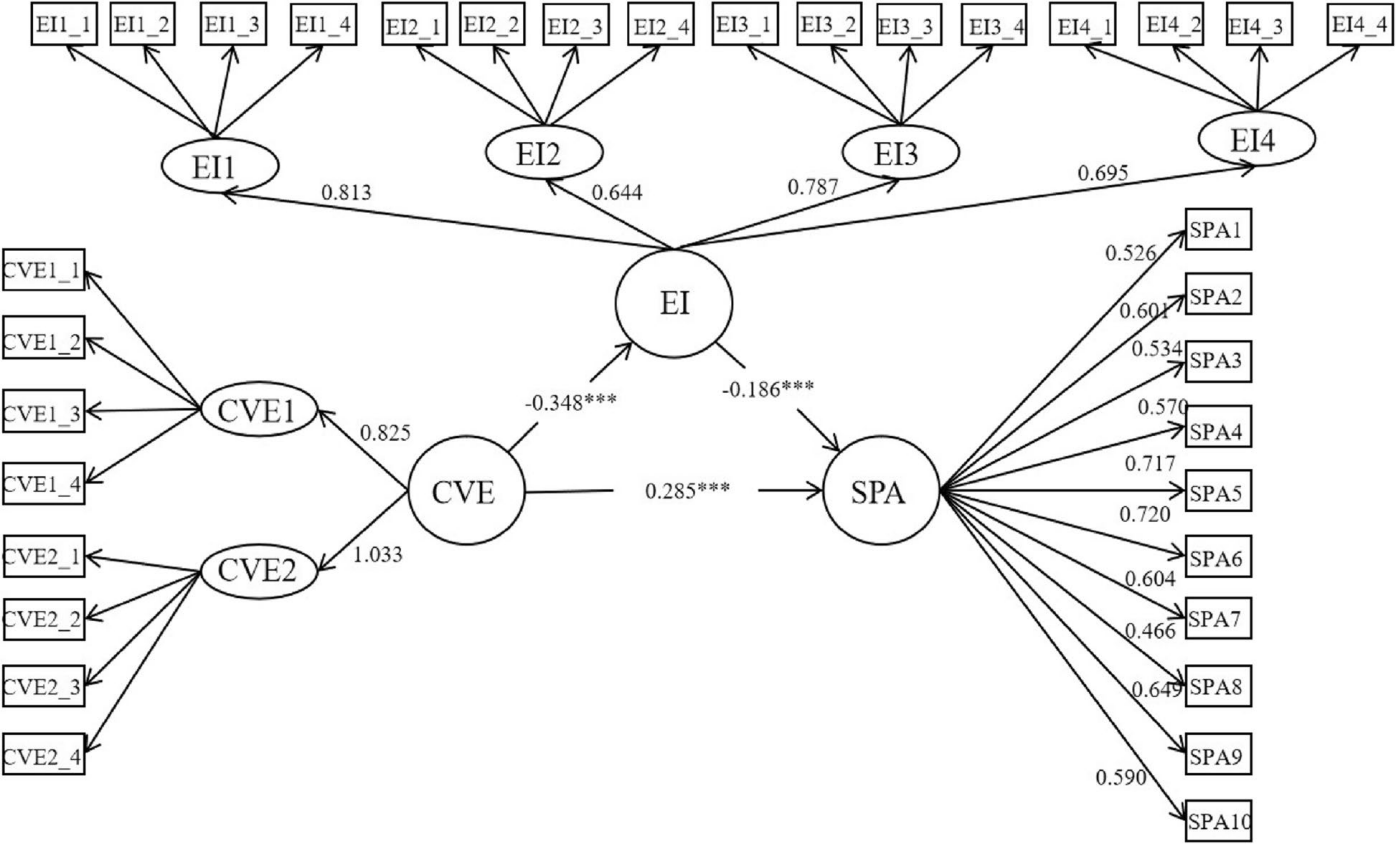
The Mediation Model. Note: *** *p* < 0.001; CVE, COVID-19 victimization experience; CVE1, catastrophic cognition; CVE2, trauma symptoms; EI, emotional intelligence; EI1, self-emotion appraisal; EI2, Others’ emotion appraisal; EI3, use of emotion; EI4, regulation of emotion; SPA, smartphone addiction

The results of the structural relationships were displayed in Fig. 2. From which, we can say that the COVID-19 victimization experiences of university students’ could significantly and positively predict their smartphone addiction (*β* = 0.285, *p* < 0.001); university students’ COVID-19 victimization experiences could significantly and negatively predict emotional intelligence (*β* = -0.348, *p* < 0.001); and emotional intelligence significantly and negatively predicted smartphone addiction (*β* = -0.186, *p* < 0.001). The result suggested that emotional intelligence exerted a partially mediating effect between university students’ COVID-19 victimization experiences and their smartphone addiction.

To examine the vigor and stability of the mediation model results, the bias-corrected non-parametric percentile bootstrap method with 5,000 times resampling was further performed. The bootstrap results were shown in Table 8. The results suggested that the indirect effect, i.e., COVID-19 victimization experiences value → emotional intelligence → smartphone addiction was 0.065 with 95% confidence interval ranging from 0.034 to 0.101, excluding 0, indicating a significant mediating effect. While the direct effect of the COVID-19 victimization experiences on smartphone addiction was 0.285, with 95% confidence interval ranging from 0.200 to 0.372, without 0, indicating a partial mediating effect. Furthermore, the total effect value, i.e. the sum of the indirect effect and the direct effect, was 0.350 with 95% confidence interval ranging from 0.272 to 0.425, precluding 0. The mediation effect accounted for 18.57% of the total effect.

**Table 8:** The results of bootstrap with 5,000 times resampling

Path	Estimated Values	95% Confidence Interval	
		lower limit	upper limit
Indirect effect: CVE → EI → SPA	0.065	0.034	0.101
Direct effect: CVE → SPA	0.285	0.200	0.372
Total effect	0.350	0.272	0.425

*CVE* COVID-19 victimization experience, *EI* emotional intelligence, *SPA* smartphone addiction

## Discussion

### Main findings

Drawing upon SCT and previous research, in a sample of 1154 university students in China, the purpose of the present study was to investigate the role of the COVID-19 victimization experience and emotional intelligence in forming university students’ smartphone addiction.

First, as is illustrated in Fig. 2, the current findings confirmed Hypothesis 1, supporting that COVID-19 victimization experiences are negative stimuli that have a significant predictive effect on smartphone addiction. This result is consistent with previous studies reporting that intolerance of uncertainty of COVID-19 correlated positively with Internet addiction [11], that experiencing occurrence of COVID-19 resulted in trauma and negative psychological symptoms [59–61], that fear of COVID-19 was found to influence smartphone addiction and mental wellbeing [8], that trauma was associated mobile phone addiction and with high insecure attachment [12], that anxiety about COVID-19 infection was the strongest predictor of smartphone addiction, followed by daily use hours [9], and risk perception of COVID-19 positively impacted internet addiction [5]. Aligning with those results, the present study further found the significant relationship between the COVID-19 victimization experience and smartphone addiction among university students.

As Chinese government have changed its zero COVID policy since December 6, 2022, more and more individuals will be infected by the COVID-19. Due to the particularity of university students, they are more likely to become the primary infected groups. Although the COVID-19 pandemic is going to be over in China and other places, it doesn’t mean that people necessarily return to their “normal” lives. That is, during the post-COVID-19 era, more and more university students will have the COVID-19 victimization experiences. In fact, university students who contracted COVID-19 would still choose to “stay at home” or keep social distancing because of the collectivistic values [62, 63]. This opens up more possibilities for university students of spending more time on new social media [64], such as smartphone. Moreover, the frequency of smartphone use increased for accessing information related to the COVID-19 pandemic and other things, which is a critical symptom of overusing and may result in the development of smartphone addiction in some individuals [65, 66]. In addition, the abundance of news about the epidemic, which was often conflicting, confusing, constantly updated and seldomly verified, brought down the tolerance levels of individuals, amplifed fear and distress, and increased the perception of threat. These factors further lead individuals to use their smartphones more intensively to cope with these feelings [35]. Thus, individuals with higher levels of COVID-19 victimization experience can result in higher levels of smartphone addiction problems among university students.

Second, the findings revealed that emotional intelligence mediated the association between COVID-19 victimization experience and smartphone addiction. That is, COVID-19 victimization experience could indirectly affect the university students’ smartphone addiction through their emotional intelligence abilities. The results are in agreement with those of previous studies indicating that emotional intelligence mediated the association between social anxiety and problematic internet use [10], between COVID-19 perceived risk and Internet addiction [46], between psychological distress and increased burnout level [43], and between perceived stress and academic performance [42]. The present study further broadens the mediating role of emotional intelligence between COVID-19 victimization experience and smartphone addiction, showing that the effects of university students’ COVID-19 victimization experience on their smartphone addiction could be compromised by their high levels of emotional intelligence abilities.

Previous studies have suggested that during the COVID-19 pandemic, individuals had more increased levels of psychological distress, anxiety, emotional exhaustion, stress, and depression than before the Covid-19 outbreak, which in turn negatively damage their emotional intelligence abilities [7, 11]. As an exceptional disaster event, individuals who contracted COVID-19 may be more likely to have negative thoughts regarding the traumatic event and feel anxious regarding their situation, which would led to lower levels of emotional intelligence. Moreover, extant studies also have revealed that increased emotional intelligence could decrease the risk of burnout, depression, psychological distress, and addictive behaviors [37, 62]. In this sense, although individuals with COVID-19 victimization experience tend to overuse their smartphones frequently, the levels of smartphone addiction could be decreased by enhancing their emotional intelligence abilities. Indeed, emotionally intelligent students, who are more capable of knowing how to appraise their own and others’ emotions consistently and deal with emotional problems effectively, are less vulnerable to developing addictive behaviors [67]. Hence, designing a special program to enhance university students’ emotional intelligence could help them to face challenges resulted from the COVID-19 victimization experiences, thus preventing them from developing smartphone addiction.

Third, the present study added further empirical evidence to support the explanation of the model of SCT. That is, the environmental factors, the individual factors and the behavioral factors is not so much isolate as could interact with each other simultaneously in a model. In this study, the mediation role of emotional intelligence (the individual factor) between the COVID-19 victimization experience (the environmental factor) and smartphone addiction (the behavioral factor) has been discovered. More specifically, the present study further discussed how COVID-19 victimization experiences affects smartphone addiction among university students during the post-COVID-19 era. The results indicated that emotional intelligence and perceived COVID-19 victimization experiences could provide a more comprehensive interpretation of smartphone addiction among university students during the epidemic. High levels of emotional intelligence were a key mediating factor for smartphone addiction among university students, which can compromise the effects of perceived COVID-19 victimization experiences on smartphone addiction.

### Practical implications

The present study could provide some practical reference for education administrators and instructors to respond to emergency public health events such as COVID-19. First, instructors should guide students to properly confront COVID-19 victimization experience and guide them to develop an proper understanding of the epidemic. For instance, education administrators should hold lectures on COVID-19 knowledge, scientifically interpret the current development of the epidemic, guide college students to accurately understand the threat to health and to have reasonable thinking in similar public health events.

Second, considered that university students’ COVID-19 victimization experiences could decrease their levels of emotional intelligence, increasing their negative emotions during the post-COVID-19 era, it is suggested that instructors should integrate emotional intelligence training programs into mental health courses so as to improve students’ emotional intelligence ability. Instructors have to help university students improve their emotion regulation ability, preclude college students from being addicted to the smartphone, and minimize the vulnerability of students to develop smartphone addiction.

Third, there may be different views on the epidemic accounts on the smartphone, which may mislead the proper understanding of the COVID-19 epidemic among university students. It is suggested that during the lockdown, education administrators should lead students to distinguish smartphone information scientifically. Instructors should advocate proper use of the smartphone and identify excessive use of the smartphone as early as possible to prevent university students from indulging smartphone for a long time, which in turn leads to smartphone addiction.

### Limitations and future research

Like other studies, some limitations should be noted so that future research could address them. First, since the present study was cross-sectional, the sample using the convenience sampling method was merely collected among the Chinese university students, it might have inferential limitations between variables. To generalize the results, therefore, a longitudinal cross-cultural study should be conducted, the sampling method and range of participants should be improved and expanded, particularly populations with cultural differences must be considered in future studies to compare differences in COVID-19 victimization experience on smartphone addiction between Chinese and other Western university students.

Second, this study used a self-report questionnaire to measure the variables, which could have resulted in socially-desirable answers. This might especially be important in relation to emotional intelligence. A note for further research is to investigate emotional intelligence with other methods, such as performance-based measures [68]. Moreover, this study do suggest that emotional intelligence on its own could have a mediating role in the relationship between COVID-19 victimization experience and smartphone addiction, but the specific subsets of emotional intelligence may have different effects. Therefore, future studies might address the mediating effects of the specific subsets of emotional intelligence separately.

Third, this study used a five-point Likert self-reported scale to measure smartphone addiction without distinguishing different types of use, and one factor loading and the AVE value of the Smartphone Addiction Scale were low than 0.5. However, the self-reported smartphone use cannot be equated with the actual use. For future studies, different types of smartphone use, e.g., process use and social usage [69], should be taken into account; to improve the validity of data and to measure smartphone use more precisely, a particular application that can track smartphone activities could also be employed.

## Conclusion

As per the SCT and previous studies, this study constructed a mediation model to explore the main influencing factors of smartphone addiction among Chinese university students during the poet-COVID-19 era. A significant positive correlation was observed among COVID-19 victimization experience (environmental factor), emotional intelligence (personal factor), and smartphone addiction (behavioral factor). SEM analysis showed that the COVID-19 victimization experience had a significant positive predictive effect on smartphone addiction and emotional intelligence of university students. Moreover, emotional intelligence played a mediating role between COVID-19 victimization experience and smartphone addiction. Considering more and more individuals are going to contract the COVID-19, more studies about the COVID-19 victimization experience and its effects should carried out.

## Data Availability

The datasets used during the current study are available from the corresponding author on reasonable request.
